# Supplementary material for: A Study of the Reliability and Accuracy of the Real-Time Detection of Forage Maize Quality Using a Home-Built Near-Infrared Spectrometer
Source: Foods. 2022 Nov 3;11(21):3490. doi: 10.3390/foods11213490 (PMC9655452; doi:10.3390/foods11213490)
Supplement: Supplementary file 1 [file foods-11-03490-s001.zip › foods-1888986-supplementary.pdf]

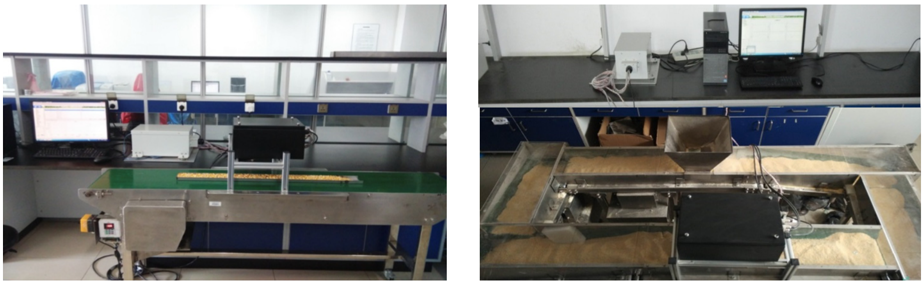

A: Home-built grating online NIR spectrometer

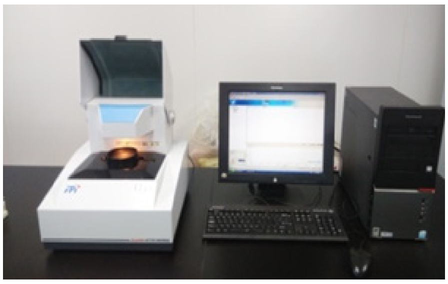

B: SupNIR-2700 grating NIR spectrometer

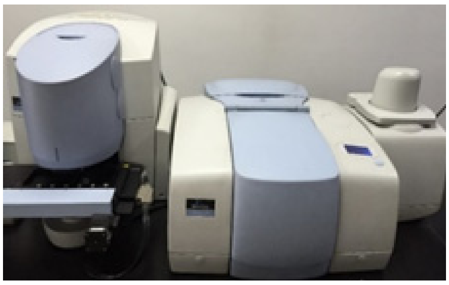

C: Spectrum 400 diffuse reflection FT NIR spectrometer

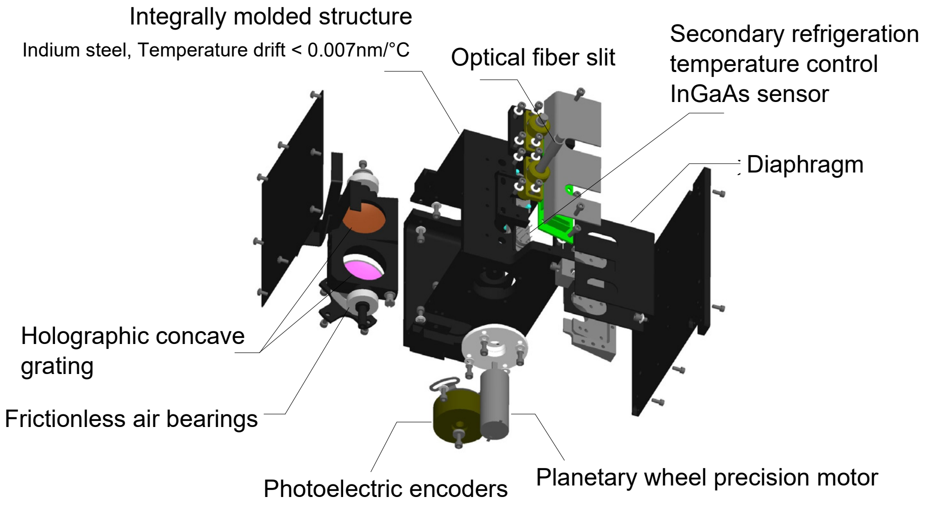

D: A schematic layout of home-built grating online NIR spectrometer

Figure S1 Three different types of near infrared spectrometers

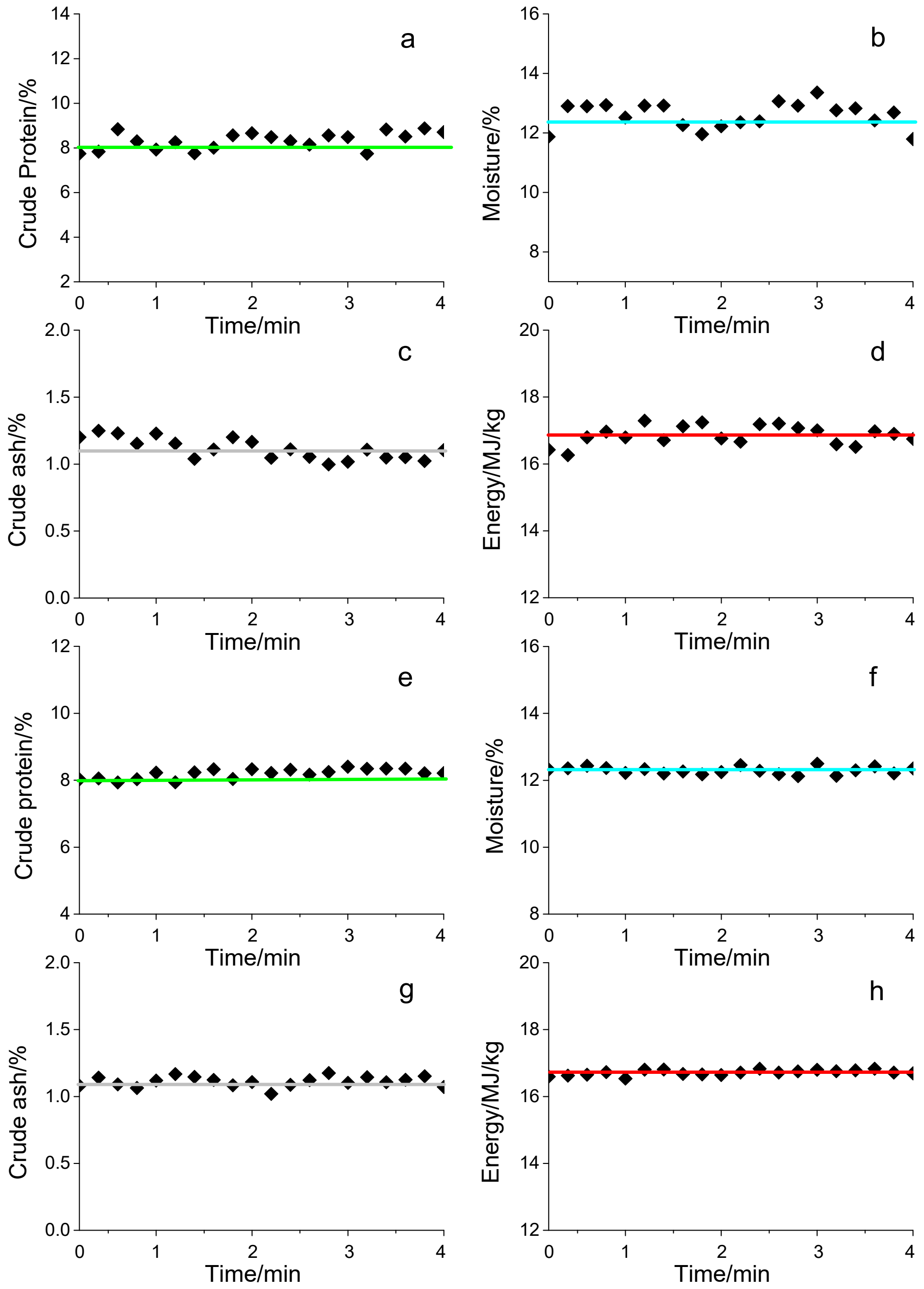

Figure S2 Repeatability of NIR online model of raw and crushed forage maize for crude protein, moisture, crude ash, and energy (a-d, raw forage maize; e-h, crushed forage maize)

Table S1 Orthogonal experimental results of influence factors of NIR online analysis

Number	Parameters			RMS
	Detection optical path /cm	Conveyor speed /cm s^{-1}	Scan times	
1	8	10	8	0.0143
2	8	20	16	0.0098
3	8	30	32	0.0065
4	12	10	32	0.0030
5	12	20	8	0.0095
6	12	30	16	0.0041
7	16	10	16	0.0057
8	16	20	32	0.0034
9	16	30	8	0.0108

Table S2 Quantitative model results of crude protein of forage maize at different detection optical path, particle size and conveyor speed

	Parameters	Calibration		Validation		
		Rc	RMSEC/%	Rp	RMSEP/%	RPD
Detection optical path /cm	8	0.93	0.29	0.84	0.39	1.65
	10	0.92	0.33	0.81	0.37	1.73
	12	0.94	0.24	0.90	0.31	2.32
	14	0.94	0.28	0.85	0.39	1.86
	16	0.94	0.27	0.86	0.41	1.62
Particle size /mm	0.425	0.96	0.22	0.96	0.17	3.78
	1	0.95	0.25	0.91	0.27	2.41
	Raw Sample	0.94	0.24	0.89	0.31	2.32
Conveyor speed /cm s^{-1}	0	0.94	0.24	0.89	0.31	2.32
	5	0.93	0.25	0.84	0.33	1.76
	10	0.93	0.29	0.78	0.40	1.56
	20	0.91	0.32	0.75	0.41	1.52

Table S3 Signal-to-noise ratio and change rate of peak area at different conveyor speed

	Conveyor speed /cm s^{-1}	5	10	20
Signal-to-noise ratio	1450 nm (Moisture)	-28.260	-11.916	-7.156
	1730 nm (Crude protein)	-50.098	-21.777	-13.531
	2250 nm (Crude lipid)	3.053	1.923	1.811
Peak area	1	-0.623	-0.600	-0.583
	2	-0.620	-0.599	-0.599
	3	-0.619	-0.593	-0.576
	4	-0.617	-0.593	-0.575
	5	-0.615	-0.589	-0.571
	6	-0.614	-0.569	-0.561
	Average	-0.618	-0.591	-0.577
	Change rate	0.003	0.011	0.013

Table S4 Result of multivariate analysis of variance

	Quadratic sum	Degree of freedom	Mean-square value	F
A	3.57089E-05	2	1.79E-05	46.848
B	4.82222E-07	2	2.41E-07	0.633
C	8.23089E-05	2	4.12E-05	107.985
SSE	0.0000	2.0000	F0.95(2, 2)=19；F0.9(2, 2)=9	
SST	0.0001	8.0000		

A: detected optical path; B: conveyor speed; C: spectra scan times.
